# A Psycholinguistic Analysis of Responses to Live-Stream Suicides on Social Media

**DOI:** 10.3390/ijerph16162848

**Published:** 2019-08-09

**Authors:** Ang Li, Dongdong Jiao, Xingyun Liu, Jiumo Sun, Tingshao Zhu

**Affiliations:** 1 Department of Psychology, Beijing Forestry University, Beijing 100083, China; 2 Institute of Psychology, Chinese Academy of Sciences, Beijing 100101, China; 3 Black Dog Institute, University of New South Wales, Sydney 2031, Australia; 4 National Computer System Engineering Research Institute of China, Beijing 100083, China

**Keywords:** live-stream suicide, psycholinguistic analysis, social media, Weibo

## Abstract

Live-stream suicide has become an emerging public health problem in many countries. Regular users are often the first to witness and respond to such suicides, emphasizing their impact on the success of crisis intervention. In order to reduce the likelihood of suicide deaths, this paper aims to use psycholinguistic analysis methods to facilitate automatic detection of negative expressions in responses to live-stream suicides on social media. In this paper, a total of 7212 comments posted on suicide-related messages were collected and analyzed. First, a content analysis was performed to investigate the nature of each comment (negative or not). Second, the simplified Chinese version of the LIWC software was used to extract 75 psycholinguistic features from each comment. Third, based on 19 selected key features, four classification models were established to differentiate between comments with and without negative expressions. Results showed that 19.55% of 7212 comments were recognized as “making negative responses”. Among the four classification models, the highest values of Precision, Recall, F-Measure, and Screening Efficacy reached 69.8%, 85.9%, 72.9%, and 47.1%, respectively. This paper confirms the need for campaigns to reduce negative responses to live-stream suicides and support the use of psycholinguistic analysis methods to improve suicide prevention efforts.

## 1. Introduction

Suicide is a major public health problem that can have harmful effects on individuals, families, and communities [1,2,3,4,5,6]. Suicidal people are often overwhelmed by feelings of hopelessness and social disconnection [7]. Therefore, the provision of crisis intervention, support, and assistance for suicidal people is essential to reduce their mortality risk [8], suggesting the importance of improving responses to suicides.

In recent years, live-stream suicide has become an emerging public health problem in many countries. On the internet, registered users are allowed to express their thoughts and feelings to a massive audience in real-time, and some of them even have used the internet platform to live stream their suicides (commonly known as live-stream suicide). Since there is not yet a consensus on the definition of live-stream suicide, in this study, it is defined as making a self-disclosure of suicidality (including suicidal ideation, suicide plans and suicide attempts) on the internet without time delays. Between 2003 and 2016, at least 193 relevant incidents have been reported in China [9]. Of these incidents, the majority took place on social media sites. When suicidal people disclose their suicidality on the internet (posting suicide-related messages), other regular users are likely to be the first to witness and respond to such suicides (posting comments on suicide-related messages). If they react appropriately, the likelihood of death may be reduced. However, a number of previous studies revealed the fact that users may react to live-stream suicides negatively. For example, Fu et al. examined a suicide incident that happened on Sina Weibo and analyzed 5971 generated posts [10]. Of these posts, 23.4% were identified as “cynical and indifferent comments”. Li et al. collected and analyzed 4969 Weibo posts with suicide-related keywords. They found that 35.42% and 5.17% of the analyzed posts were associated with stigmatizing attitudes and negative behavioral intentions (e.g., refusing to offer help and encouraging suicide), respectively [11]. Ma et al. investigated six suicide incidents, and recognized “making cynical or indifferent comments, ‘like′ and incitement” as a major type of the response [12]. O′Dea et al. explored the first replies to suicide-related posts on Twitter and found that 23% of them reflected “dismissive or encouraging of the suicide” [13]. Results of these studies suggest that it is necessary to filter out such negative responses to reduce the likelihood of suicide death. However, users who witness live-stream suicides often post thousands of comments on suicide-related messages within several hours after disclosure of suicidality. The sheer volume of data makes it difficult for human coders to determine whether each comment reflect negative expressions or not. Therefore, there is a dire need for automatic detection of negative expressions in responses to live-stream suicides.

An examination of the words that people use in everyday language can provide insight into their psychological profiles [14]. A few recent studies found psycholinguistic characteristics of stigmatizing expressions in social media posts [15,16], which suggests that the use of psycholinguistic analysis methods may improve our ability to automatically detect negative responses to mental health problems. However, these previous studies were limited by insufficient data collection and inadequate investigation of negative responses. Specifically, in these studies, researchers searched data using a set of keywords. As a result, such collected data may only reflect general opinions on a certain topic rather than actual responses. Moreover, researchers focused solely on stigmatizing attitudes, and dismissed other types of negative responses (e.g., negative behaviors/behavioral intentions). Therefore, additional analyses are necessary.

This study aims to examine actual responses to live-stream suicides on social media (Sina Weibo) and proposes the use of psycholinguistic analysis methods to establish computational models for automatic detection of negative expressions in responses.

## 2. Materials and Methods

**Figure 1:**
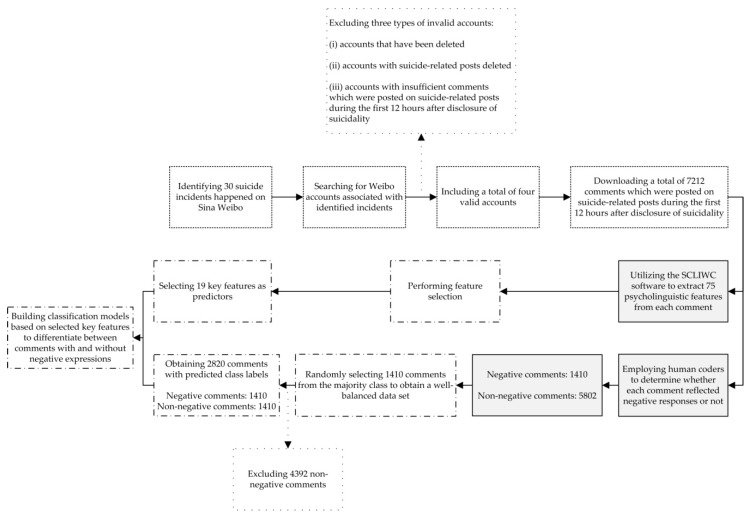
Research process.

The study was conducted in accordance with the Declaration of Helsinki. The protocol was approved by the Institutional Review Board at the Institute of Psychology, Chinese Academy of Sciences (protocol number: H09036 and H15009). The research process consisted of the following steps Figure 1: (i) Data collection, (ii) data pre-processing, and (iii) data analysis. Participant informed consent was not required for analyzing publicly available information [17,18]. To protect the participant privacy, personally identifiable information (including usernames and real names) was excluded from the analysis.

### 2.1. Data Collection

First, four incidents of live-stream suicides, which took place on Sina Weibo, were selected. Similar to Twitter, Sina Weibo is a free social media site that enables registered users to communicate and interact with others in real-time using posts. Although some users opt to privatize their accounts, the majority of the Weibo content is publicly available for viewing and downloading. To easily access and analyze data, in this study, we only focused on Sina Weibo rather than other internet platforms. According to a previous study [9], 30 relevant incidents that took place on Sina Weibo were identified. After that, the Weibo accounts associated with identified incidents were searched and found. To collect reliable and valid data, a further scrutiny of the Weibo accounts occurred to exclude the following: (i) Accounts that have been deleted, (ii) accounts with suicide-related posts deleted, and (iii) accounts with very few comments which were posted on suicide-related posts during the first 12 h after disclosure of suicidality (i.e., release of the first suicide-related post). As a result, a total of four target accounts were included for further analysis Table 1.

Second, for each target account, the corresponding comments were downloaded and analyzed. Since the duration of suicidal crises could be extended over several hours [19,20,21], in this study, responses were recognized as commenting on suicide-related posts during the first 12 h after release of the first suicide-related post. Finally, a total of 7212 comments were obtained. 

### 2.2. Data Pre-Processing

First, to get predicted class labels for data modeling, two human coders were recruited to investigate the nature of each comment (negative responses or not). The coding framework was developed on the basis of expert consensus and available evidence. In specific, one researcher reviewed a number of relevant studies [10,11,12,13] and performed an inductive analysis of all 7212 comments to construct an initial framework. After that, two recruited coders gained a deep understanding of the initial framework and gave suggestions for its amendment. Finally, the initial framework was amended accordingly, and the formal framework was established in Table 2. By using the formal framework, two coders analyzed all 7212 comments independently. The levels of coding consistency between coders was evaluated by computing Cohen′s *κ* coefficients. If any inconsistency arises, a third researcher′s opinion can resolve this issue.

Second, to get predictors for data modeling, the simplified Chinese version of the LIWC software (SCLIWC) (developed by the Computational Cyber-Psychology Lab at the Institute of Psychology, Chinese Academy of Sciences; website: http://ccpl.psych.ac.cn/textmind/) was utilized to extract psycholinguistic features from each comment. SCLIWC is a reliable and valid text analysis tool, which can be used to automatically estimate the words frequency in different psycholinguistic categories (e.g., emotional, cognitive, and other structural categories) [22]. Therefore, a total of 75 psycholinguistic features can be obtained for each comment.

### 2.3. Data Analysis

To filter out negative responses that may be harmful to suicidal users, in this study, all 7212 comments were classified into two groups, including the negative group (i.e., comments related to “making negative responses”) and non-negative group (i.e., comments related to any one of other four categories). The WEKA software (Version 3.8.1) was utilized to build a series of classification models to differentiate between comments reflecting negative and non-negative responses.

First, to solve the class imbalance problem, a certain number of comments were randomly selected from the pool of the non-negative group. In this study, the data set is heavily imbalanced with 80% of data points being non-negative (negative group: 1410 comments; non-negative group: 5802 comments). In other words, in a binary classification problem, when 80% of data points belong to the false class (non-negative responses), a default prediction of false for all data points would lead to a classifier with high accuracy (80%), even though the classifier has not learnt anything about the classification problem. Therefore, by using the method of simple random sampling, a total of 1410 comments (providing social support: 1190 comments; calling for help: 55 comments; expressing shock: 38 comments; unspecified: 127 comments) were randomly selected from the majority class (non-negative group) for further analysis. Between the sample (selected 1410 non-negative comments) and the population (all 5802 non-negative comments), there were no significant differences in the proportion of cases across the four non-negative subcategories. As a result, a well-balanced data set was obtained (negative group: 1410 comments; non-negative group: 1410 comments).

Second, to maximize the modeling performance, a series of key psycholinguistic features were selected as predictors. Since there is not yet a consensus on the selection of key psycholinguistic features, in this study, three different feature selection methods were used to explore key features automatically, including the gain ratio attribute evaluator (GRAE), the significance attribute evaluator (SAE), and the Chi-squared attribute evaluator (CAE). Based on such three methods, the weight of each feature was evaluated by measuring the gain ratio in terms of the class (GRAE), computing the probabilistic significance as a two-way function (feature-classes and classes-feature association) (SAE), and estimating the value of the Chi-squared statistic in terms of the class (CAE), respectively. As a result, according to different feature selection methods, all 75 features can be ranked with respect to their individual evaluations. In this research, key features were recognized as features ranked among the top 25 by all three methods (i.e., GRAE, SAE, and CAE). Key features were selected for use in the model construction.

Third, by using different algorithms (simple logistic regression, SLR; multilayer perceptron neural networks, MLPNN; support vector machine, SVM; random forest, RF), four classification models (SLR, MLPNN, SVM, and RF models) were established based on selected key features. 10-fold cross-validation was used to test each model. Specifically, the data set was randomly divided into ten subgroups with the same sample size. Each subgroup was used to test the model which was built on the other nine subgroups. After ten rounds of model training, the modeling results were integrated into a final model. Since coefficients in three of the four models (i.e., MLPNN, SVM, and RF models) could not indicate linear relationships between predictors and predicted class labels, regression coefficients in the SLR model were estimated to discover such relationships.

In this study, three indicators were used to measure the classification performance, including precision (number of true positives/number of instances predicted to be positive), recall (number of true positives/number of positive instances), and F-measure (a tradeoff between precision and recall).

Furthermore, an additional indicator (i.e., screening efficacy) was computed to evaluate the performance in reducing the workload of human coders for searching negative responses. In this study, the value of the screening efficacy can be estimated like this: (number of instances - number of instances predicted to be positive)/number of instances. For example, among 100 comments, 70 comments were predicted to be negative responses. Therefore, the value of the screening efficacy should be 30%, implying that 30% of the workload of human coders can be reduced. In other words, only 70% of comments need to be analyzed by human coders.

## 3. Results

### 3.1. Coding

The outcomes of the coding task were shown in Table 3. Across different suicide incidents, the Cohen′s *κ* coefficients ranged from 0.83 to 0.89, indicating a satisfying level of agreement [23].

Results showed that the proportions of comments reflecting positive responses (i.e., comments related to “providing social support” or “calling for help”) ranged from 46.38% to 80.97%, with an average of 69.93%; while the proportions of comments reflecting negative responses ranged from 7.32% to 42.66%, with an average of 19.55%.

### 3.2. Differentiating between Negative/Non-Negative Responses

A total of 19 key features were selected in Table 4. The highest values of precision, recall, F-measure, and screening efficacy reached 69.8%, 85.9%, 72.9%, and 47.1%, respectively as shown in Table 5. The logistic regression coefficients were shown in Table 6. Compared with non-negative comments, negative comments were related to an increased use of words in categories of Impersonal Pronouns (*β* = 1.05), Conjunctions (*β* = 3.15), Affective Processes (*β* = 0.83), Negative Emotion (*β* = 1.66), Exclusive (*β* = 2.28), and Death (*β* = 4.47); and were related to a decreased use of words in categories of Inclusive (*β* = −0.99) and Assent (*β* = −1.66).

## 4. Discussion

In this paper, by using psycholinguistic analysis methods, we analyzed responses to live-stream suicides on Sina Weibo and discovered the ways in which negative responses are presented on social media.

First, there is a dire need for campaigns to reduce negative responses to live-stream suicide. Results of this study showed that, despite many positive comments, a large number of negative comments existed. According to the current study, the average proportion of negative comments reached 19.55%, which is consistent with results of other previous studies (≈23%) [10,13]. Reducing suicide stigma and enhancing public awareness of suicide literacy may be the key to improving reactions to live-stream suicides fundamentally [11,16]. Social media campaigns might be helpful to address this concern. Specifically, social media enables registered users to bring personal experiences into the public domain, which might influence public attitudes, mainstream media, and even policy [24], such as the “Stigma Watch” program (https://www.sane.org/stigmawatch). Moreover, social media users are allowed to create their own social networks, which can be leveraged to facilitate the acceptance of received knowledge and then accelerate the change of individual attitude and behavior [25,26,27].

Second, the use of psycholinguistic analysis methods facilitates automatic detection of negative expressions in responses to live-stream suicides on social media. In this study, by using psycholinguistic analysis methods, computational models were established to differentiate between comments with and without negative expressions automatically. Results showed that the highest F-measure value reached 72.9%. Compared with results of similar studies (F-measure: 37–83%) [15,16,18,28,29,30], the classification performance is satisfying in this current study. Moreover, with the help of established classification models, the workload of human coders can also be reduced considerably (screening efficacy: 31.7%–47.1%).

Third, the use of psycholinguistic analysis methods provides insight into how negative responses are presented on social media. According to the coding framework, in this study, negative responses included expressing negative attitudes (expressing cynical, dismissive, and indifferent attitudes) and manifesting negative behaviors/behavioral intentions (refusing to offer help and encouraging suicide). Results showed that negative responses were associated with an increased use of words in categories of Impersonal Pronouns (e.g., it, those), Conjunctions (e.g., but, whereas), and Exclusive (e.g., without, exclude), and were associated with a decreased use of words in the category of Inclusive (e.g., with, include), indicating a desire to make a distinction between people in one group and people from the other group [14,31,32]. Furthermore, negative responses were also related to an increased use of words in categories of Affective Processes (e.g., cried, abandon) and Negative Emotion (e.g., ugly, nasty), indicating a high level of negative emotion. Such psycholinguistic patterns might fit into two major elements of stigmatizing attitudes, including cognitive separating and emotional reactions [33], which is consistent with results of previous studies [15,16]. In addition, a decreased use of Assent-related words (e.g., agree, yes) indicated negative responses contained fewer positive assertions than non-negative responses. Apart from expressing negative attitudes, manifesting negative behaviors/behavioral intentions is another major type of the negative response. It might be the reason why negative responses were related to an increased use of words in the category of Death (e.g., kill, bury).

Limitations exist. First, only four suicide incidents were involved in this study. Although a large number of comments, the small number of incidents may limit the generalizability of the current study findings. Second, given that a number of live-stream suicides happened outside of Sina Weibo, different outcomes may be found on other internet platforms. Third, there is no evidence to confirm that responses to live-stream suicides are the same as responses to offline suicides. Fourth, social media users are not representative of the general population, and thus the outcomes may not be applicable to the general public. Finally, it is unclear if the findings in this research can also be applied to other types of language. In spite of these disadvantages, in this study, a non-intrusive method was used to analyze responses to live-stream suicides. As a result, the current research should have high ecological validity and is possible to indicate the actual reactions to live-stream suicides. In addition, this study did not just focus on what a comment says precisely, but how it is expressed. Therefore, the use of psycholinguistic analysis methodologies can be beneficial to enhance the validity of automatic detection of negative responses.

## 5. Conclusions

This paper confirms the need for campaigns to reduce negative responses to live-stream suicides and supports the use of psycholinguistic analysis methods to improve suicide prevention efforts.

## Figures and Tables

**Table 1:** Details of suicide incidents.

	Incident 1	Incident 2	Incident 3	Incident 4
Time	12/07/2012 21:05	16/02/2013 23:57	09/12/2013 17:36	19/02/2016 19:57
Location	Sichuan	Beijing	Shandong	Shanghai
Gender	Female	Female	Female	Male
Age	31	31	33	40
Suicide method	Taking poisons & charcoal burning	Jumping from a high place	Drowning	Hanging
Suicide cause	Relationship breakup	Mental illness	Work-related stress	Mental illness
Result	Rescued	Died	Died	Died

**Table 2:** Coding framework.

Category	Definition	Example Weibo Post
Providing social support	Expressing care and compassion, or providing information, advice, and resources	“I hope you are ok! Don′t die” “You should get professional help now ...” “My mom is an expert in this field! Don′t die!! I can ask her to help you!!!”
Calling for help	Calling police and other users for help	“Call the police!” “Is there anyone who knows his address and can go to his place to help him? ...”
Expressing shock	Feeling surprised and upset	“What′s up, don′t scare me”
Making negative responses	Expressing cynical, dismissive, and indifferent attitudes, or refusing to offer help and encouraging suicide	“Is there anything wrong with your brain? Why not to kill yourself quietly ...” “It is her last wishes, Why do you want to stop her” “If you want to kill yourself, please be quick ...”
Unspecified	Replying with unspecified intent or meaning	“ 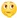 ” “.........” “Sigh”

**Table 3:** Coding results.

Category	Incident 1	Incident 2	Incident 3	Incident 4	Sum
Providing social support	671 (41.06%)	1583 (79.75%)	912 (59.07%)	1640 (80.04%)	4806 (66.64%)
Calling for help	87 (5.32%)	20 (1.01%)	111 (7.19%)	19 (0.93%)	237 (3.29%)
Expressing shock	41 (2.51%)	37 (1.86%)	28 (1.81%)	67 (3.27%)	173 (2.40%)
Making negative responses	697 (42.66%)	192 (9.67%)	371 (24.03%)	150 (7.32%)	1410 (19.55%)
Unspecified	138 (8.45%)	153 (7.71%)	122 (7.90%)	173 (8.44%)	586 (8.13%)

**Table 4:** Psycholinguistic features selected by different methods.

	Gain Ratio Attribute Evaluator	Significance Attribute Evaluator	Chi-Squared Attribute Evaluator
1	Total Pronouns	Total Pronouns	Total Function Words
2	Adverbs	Affective Processes	Affective Processes
3	Cognitive Processes	Cognitive Processes	Adverbs
4	Death	Adverbs	Total Pronouns
5	Affective Processes	Exclusive	Cognitive Processes
6	Total Function Words	Biological Processes	Biological Processes
7	Exclusive	Death	Exclusive
8	Biological Processes	Total Function Words	Death
9	Body	Body	Auxiliary Verbs
10	Impersonal Pronouns	Impersonal Pronouns	Impersonal Pronouns
11	Common Verbs	Auxiliary Verbs	Conjunctions
12	Conjunctions	Assent	Body
13	Auxiliary Verbs	Conjunctions	Common Verbs
14	Fillers	Common Verbs	Personal Pronouns
15	Family	Personal Pronouns	Positive Emotion
16	Swear Words	Fillers	Assent
17	Third Pers Plural	Negative Emotion	Second Pers Singular
18	Anger	Relativity	Negative Emotion
19	Personal Pronouns	Family	Prepositions
20	Negative Emotion	Second Pers Singular	Inclusive
21	Assent	Inclusive	Discrepancy
22	Nonfluencies	Humans	Health
23	Inclusive	Positive Emotion	Relativity
24	Social Processes	Social Processes	Humans
25	Humans	Swear Words	Social Processes

*Note.* Features that dropped out of the top 25 were not listed.

**Table 5:** Performance of classification models in detecting negative responses.

	Precision	Recall	F-Measure	Screening Efficacy
Simple Logistic Regression	68.8%	72.8%	70.7%	47.1%
Multilayer Perception Neural Networks	66.2%	79.6%	72.3%	39.9%
Support Vector Machine	62.9%	85.9%	72.6%	31.7%
Random Forest	69.8%	76.2%	72.9%	45.4%

**Table 6:** Regression coefficients in the Simple Logistic Regression model.

	Predictors	*β*
Negative responses (1) vs. Non-negative responses (0)	Total Function Words	2.31
	Total Pronouns	0.30
	Impersonal Pronouns	1.05
	Common Verbs	−0.62
	Auxiliary Verbs	0.54
	Adverbs	−0.24
	Conjunctions	3.15
	Humans	2.21
	Affective Processes	0.83
	Negative Emotion	1.66
	Inclusive	−0.99
	Exclusive	2.28
	Biological Processes	−0.16
	Body	5.19
	Death	4.47
	Assent	−1.66

***Note*.** Predictors without estimated *β* values were not listed.
